# Statin utilization and cardiovascular outcomes in a real-world primary prevention cohort of older adults

**DOI:** 10.1016/j.ajpc.2024.100664

**Published:** 2024-04-05

**Authors:** Aaron J. Walker, Jianhui Zhu, Floyd Thoma, Oscar Marroquin, Amber Makani, Martha Gulati, Eugenia Gianos, Salim S. Virani, Fatima Rodriguez, Steven E. Reis, Christie Ballantyne, Suresh Mulukutla, Anum Saeed

**Affiliations:** a Florida State University College of Medicine, Tallahassee, FL, United States; b University of Pittsburgh Medical Center, Pittsburgh, PA, United States; c Cedars-Sinai Medical Center, Los Angeles, CA, United States; d Northwell Health, New York, NY, United States; e Baylor College of Medicine, Houston, TX, United States; f Stanford Health Care, Stanford, CA, United States

**Keywords:** Statin, Older adults, Primary prevention, ASCVD

## Abstract

**Background:**

Statins are a cost-effective therapy for prevention of atherosclerotic cardiovascular disease (ASCVD). Guidelines on statins for primary prevention are unclear for older adults (>75 years).

**Objective:**

Investigate statin utility in older adults without ASCVD events, by risk stratifying in a large healthcare network.

**Methods:**

We included 8,114 older adults, without CAD, PVD or ischemic stroke. Statin utilization based on ACC/AHA 10-year ASCVD risk calculation, was evaluated in intermediate (7.5%-19.9%) and high-risk patients (≥ 20%); and categorized using low and ‘moderate or high’ intensity statins with a follow up period of ∼7 years. Cox regression models were used to calculate hazard ratios for incident ASCVD and mortality across risk categories stratified by statin utilization. Data was adjusted for competing risk using Elixhauser Comorbidity Index.

**Results:**

Compared with those on moderate or high intensity statins, high-risk older patients not on any statin had a significantly increased risk of MI *[HR 1.51 (1.17–1.95); p<0.01]*, stroke *[HR 1.47 (1.14–1.90); p<0.01]* and all-cause mortality *[HR 1.37 (1.19–1.58); p<0.001]* in models adjusted for Elixhauser Comorbidity Index**.** When comparing the no statin group versus the moderate or high intensity statin group in the intermediate risk cohort, although a trend for increased risk was seen, it did not meet statistical significance thresholds for MI, stroke or all-cause mortality.

**Conclusion:**

Lack of statin use was associated with increased cardiovascular events and mortality in high-risk older adults. Given the benefits appreciated, statin use may need to be strongly considered for primary ASCVD prevention among high-risk older adults. Future studies will assess the risk-benefit ratio of statin intervention in older adults.

## 1 Introduction

Atherosclerotic cardiovascular diseases (ASCVD) are the leading cause of morbidity and mortality globally [1]. In the United States, ASCVD related morbidity and mortality accounts for over 200 billion dollars annually in healthcare services [1]. The United States demographics is shifting towards an older population and with this comes a sizeable increase in cardiovascular disease incidence, prevalence, morbidity, mortality and downstream economic burden [2,3]. Stroke from individuals > 85 years of age made a large percentage (17%) of the total strokes in the United States [4].

Statins are a well-studied drug class that have proven to be a cost-effective solution to reducing ASCVD risk for primary prevention of ASCVD events [1,5,6]. Multi-societal guidelines including the American College of Cardiology (ACC)/American Heart Association (AHA) guidelines on the Primary Prevention of Cardiovascular Disease (2019) [1], US Preventive Services Task Force statement (2022) [7], Canadian Cardiovascular Society (2021) [8], and European Society of Cardiology/European Atherosclerosis Society (2019) [9], state that statin therapy should be used as first line therapy in specific risk groups. However, the major statin guidelines have unclear recommendations on statin initiation and/or statin intensity for primary prevention in older adults >75 years of age.

The ACC/AHA pooled cohort equations (PCE) derived risk score assists in guiding the use of statin therapy in adults for primary prevention [10]. However, the PCE does not estimate risk for a proportion of older adults who are over 79 years of age, thus cannot be used to determine the statin recommendation. A study attempted to use other indicators outside of a ASCVD 10-year risk to determine if primary prevention should be offered to older adults > 75 years of age, such as using the coronary artery calcium (CAC) score but this was not entirely conclusive on whether CAC score can assist in guiding the decision to initiate statin treatment for primary prevention in older adults [11]. Limited data on the utilization of statin therapy in real-world primary prevention cohorts has been previously reported [12], however, such knowledge remains scarce for the vulnerable and high risk population of adults ≥75 years of age.

In the current study, we examined the utilization of statin therapy in a contemporary cohort of older adults aged 75 years and older, for primary prevention of ASCVD events stratified by their baseline 10-year ASCVD risk. Further, we evaluated the impact of statin therapy on ASCVD events and mortality.

## 2 Methods

### 2.1 Research ethics statement

This study was approved by a Quality Improvement and Institutional Review Board committees. The informed consent requirement was waived for this study since it was restricted to secondary data analysis.

### 2.2 Population cohort

Detailed methodology of the cohort selection and descriptions have previously been published [12]. Briefly, the University of Pittsburgh Medical Center (UPMC) is a large multihospital health network located in Western Pennsylvania consisting of over 35 hospitals and over 400 outpatient clinics. Included in the analysis were patients with at least 2 health care interactions at a UPMC facility between January 2013 and December 2017 who had at least one lipid result within 180 days of the first health care system interaction (index visit). Through a system integrated into UPMC's Clinical Data Warehouse, data on medical care was obtained via clinical databases, structured administrative tools and an electronic health record (EHR). The baseline date of entry to the cohort was determined by the date of the indexed UPMC facility visit. At least one baseline covariates data was available in the system for review for all patients included in the study.

Individuals were excluded if they had baseline history of cardiovascular disease, defined as the presence of a code for any of the following[13]: stroke or cerebrovascular disease, ischemic stroke, peripheral vascular disease, and transient ischemic attack (*International Classification of Diseases, Ninth Revision [ICD-9] and Tenth Revision [ICD-10]* codes) or[6] coronary artery disease, including myocardial infarction, angina, and revascularization, and[10] congestive heart failure (*ICD-9*). Patients in hospice or a skilled nursing facility or those who had a history of rhabdomyolysis were also excluded from the study. Individuals were also excluded if they were outside the age range of 75–79 years of age.

The most common reasons for exclusion were age, presence of history of ASCVD, absence of baseline missing variables, and absence of follow-up data. Missing values (in total cohort 1.01–3.14%) were treated by imputed mean value of missing variable in each group.

### 2.3 Covariates and PCE-Derived risk

Baseline characteristics included sociodemographic data (including age, sex, and race/ethnicity and Elixhauser comorbidity score calculation which is a set of 30 comorbidity indicators) [14], low-density lipoprotein cholesterol, and PCE variables including total cholesterol, high-density lipoprotein cholesterol, treatment for hypertension, systolic blood pressure, current smoking status, and diabetes. Administrative data sources within the UPMC Clinical Data Warehouse were used to derive demographic variables, such as age (difference between the index date and date of birth), race (categorized as Black or White), sex, and medication use.

Diabetes mellitus was defined as the use of antihyperglycemic agents before the baseline date plus either 2 *ICD-9* or *ICD-10* diagnosis codes. Blood pressure treatment was defined as an active prescription on the baseline date for at least one of the following medication classes, taken as a single agent or in combination formulations: diuretics, angiotensin-converting enzyme inhibitors, angiotensin II receptor inhibitors, α-blockers, β-blockers, and calcium channel blockers. Consistent with the PCE, smoking history was categorized as current or not current (a composite of never and former smoking).

The primary prevention cohort was divided into categories of PCE-derived 10-year risk [10], that is, intermediate risk (7.5%–19.9%) and high risk (≥20%). Low-risk and borderline risk categories were not used as age ≥ 75 years of age made all individuals at least intermediate risk. Per the ACC/AHA cholesterol guidelines of 2013, guideline-directed statin intensity (GDSI) for the intermediate or high-risk group 75 years of age or less was a moderate or high intensity statin. For older adults greater than 75 years of age no guideline specified statin intensity exists, so patients were categorized into using no statin, low intensity statin and ‘moderate or high’ intensity statins.

The statin used by the patients were defined as either high intensity, which included atorvastatin 40 and 80 mg, rosuvastatin 20 and 40 mg; moderate intensity; atorvastatin 10 and 20 mg, rosuvastatin 5 and 10 mg, simvastatin 20 and 40 mg, pravastatin 40 and 80 mg, lovastatin 40 and 80 mg; low intensity; simvastatin 10 mg, pravastatin 10 and 20 mg, lovastatin 20 mg, fluvastatin 20 and 40 mg. Due to the limited use of pitavastatin among our cohort, patients on this medication were excluded from the analysis.

### 2.4 Outcomes

The primary outcomes included time to a moderate or high intensity statin in older adults ≥ 75 years of age per ASCVD risk category, incident coronary artery disease including myocardial infarction and revascularization, incident stroke (transient ischemic attacks and/or ischemic stroke) events and mortality. Time to ‘moderate or high’ intensity statins was defined as the time, in years, from first interaction with the health care system to achieving at least a moderate intensity statin. Outcomes were followed from their baseline visit until March 2020. This study used a follow up period of ∼ 7 years.

The Social Security Death Index was used to assess mortality. Our health care system is exempt from the 3-year delay period by the Social Security Administration.

All medical records were reviewed for combination of ICD codes from the Center for Medicare & Medicaid Services data sources and documentation review.

### 2.5 Statistical analysis

Range of data points such as the minimum and maximum values as well as measures of central tendency including both median, and mean were analyzed for all baseline descriptive statistics included in the study to detect outliers and missing values. Missing data points were rare, but when this incident occurred the missing value was replaced by the simple mean imputation across the risk groups. Descriptive characteristics with continuous variables are presented as mean and SD with the difference of mean across the ASCVD groups is assessed by 1-way ANOVA. Descriptive characteristics with categorical variables are presented as frequencies and proportions with the difference of frequencies compared using the χ^2^ test.

Cox proportional hazards regression models adjusted for age, gender, race, high density lipoprotein cholesterol, low density lipoprotein cholesterol, systolic blood pressure, current smoker and no diabetes, were used to compare the hazard ratios of primary outcomes among each risk category and between use of ‘moderate or high’ intensity statins versus low intensity statin and no statin use before the first outcome of interest. Regression models were fully adjusted for burden of conditions limiting life-expectancy using the van Walraven algorithm for the Elixhauser Comorbidity [15]. The van Walraven algorithm for the Elixhauser Comorbidity Index uses 21 of the 30 comorbidity indicators in the original Elixhauser comorbidity score to assist in adjusting for confounding [14,15]. These comorbidity indicators include but are not limited to renal failure, liver disease, lymphoma, a solid tumor without metastasis and metastatic cancer [15]. The statin intensity category variable was treated as time invariant, and classification of ‘moderate or high’ or statin category was based on the statin status before each outcome. Individuals were censored between 2 and 7 years after baseline in the corresponding analysis if they had not previously experienced the primary outcome. The time to ‘moderate or high’ intensity statins across the ASCVD risk groups was estimated using Kaplan-Meier method. Additionally, we also used “no statin use” as a reference category to calculate HRs for outcomes.

All analyses were completed using SAS version 9.4 software (SAS Institute). Statistical significance was set at α =0.05. All tests of statistical significance were 2-tailed.

## 3 Results

**Fig. 1:**
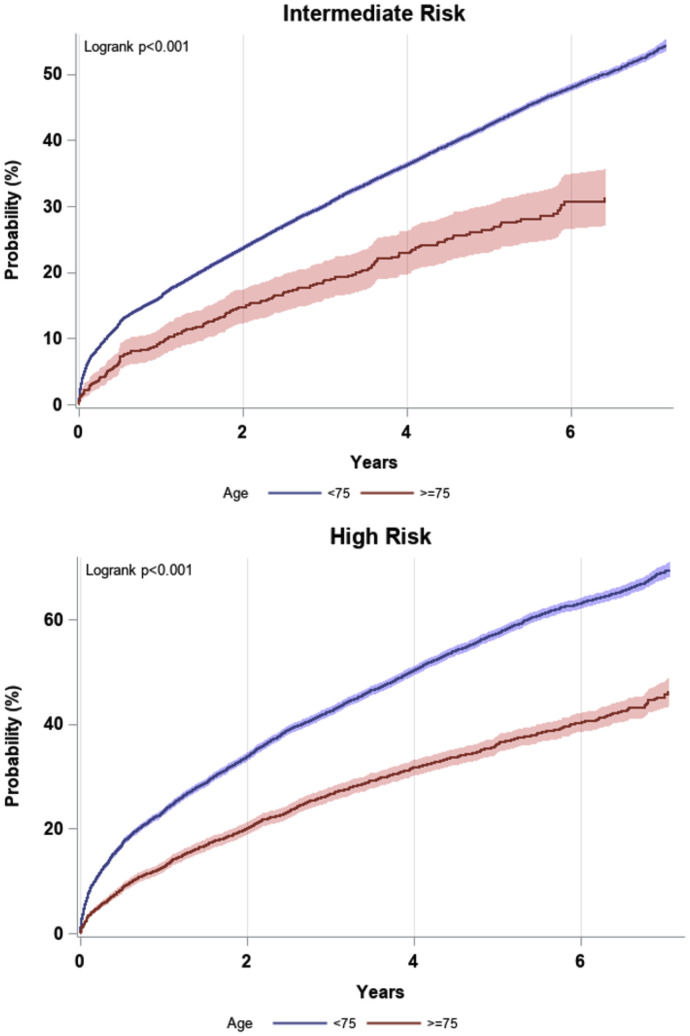
KM Estimated Probability of Patients Starting GDSI (<75 Years of Age) Or At Least a Moderate Intensity Statin (≥ 75 Years of Age) After Enrolled.

Fig. 1 in the Data Supplement illustrates the cohort creation of 2348,822 patients who were treated at a UPMC facility from between January 2013 and December 2017 [12], a total of 8114 (0.35%) were eligible after implementing the inclusion and exclusions criteria.

The 10-year ASCVD risk categories were defined as intermediate-risk (7.5%–19.9%), and high-risk (≥20%). Statins are defined as high intensity: Atorvastatin 40 and 80 mg, Rosuvastatin 20 and 40 mg; moderate intensity: Atorvastatin 10 and 20 mg, Rosuvastatin 5 and 10 mg, Simvastatin 20 and 40 mg, Pravastatin 40 and 80 mg, Lovastatin 40 and 80 mg.Abbreviations: ASCVD, atherosclerotic cardiovascular disease.

**Figure:**
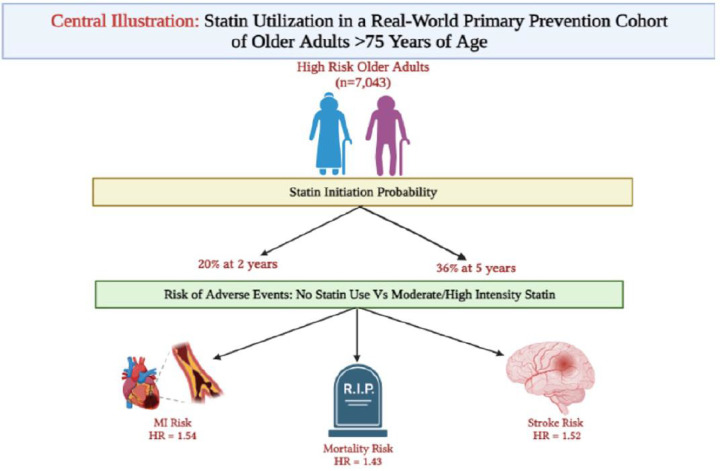
Central Illustration: Statin Utilization in a Real-World Primary Prevention Cohort of Older Adults >75 Years of Age, The high-risk 10-year ASCVD risk category was defined as a risk of ≥20%. Statins are defined as high intensity: Atorvastatin 40 and 80 mg, Rosuvastatin 20 and 40 mg; moderate intensity: Atorvastatin 10 and 20 mg, Rosuvastatin 5 and 10 mg, Simvastatin 20 and 40 mg, Pravastatin 40 and 80 mg, Lovastatin 40 and 80 mg. *Abbreviations: HR; hazard ratios*.

In this study among 8114 patients between 75 and 79 years (66% women and 34% men), 1071 (13.2%) were categorized as intermediate risk and 7043 (86.8%) as high risk (Table 1). The mean age of the cohort was 76.9 ± 1.19 years of age. The use of any statin was 36.5% in the intermediate risk group and 46.1% in the high-risk group. We also noticed the 45% aspirin use in the high-risk group is increased compared to 33% in the intermediate risk group. The prevalence of smoking was significantly higher (p-value <0.001) in the high-risk group (8.2%) versus the intermediate-risk group (1.3%). This trend was also seen with the prevalence of hypertension medication use between the high-risk group (57.5%) versus the intermediate-risk group (25.7%). The same significance was also seen between the high-risk versus intermediate-risk groups for BMI (29.2 ± 5.64 versus 27.4 ± 5.37 respectively), aspirin use (45.1% versus 33.3% respectively) and diabetes (25.2% versus 0.37% respectively) (Table 1). Most patients in the cohort were seen in their primary care physician (PCP) office (85.3%), with only 10% of intermediate-risk individuals and 15.5% of high-risk individuals being seen in cardiology offices. Statin use of any intensity was 36.5% among the intermediate-risk cohort and 46% among the high-risk cohort (Table 1).

**Table 1:** Baseline Characteristics of Population Aged 75–79 Categorized by ASCVD Risk.

Baseline characteristics	Total Population (8114)	ASCVD risk category (n)	
		Intermediate Risk (1071)	High Risk (7043)
Age	76.9 ± 1.19	76.3 ± 1.04	77.0 ± 1.18
Male	34.08%	7.56%	38.11%
Female	65.92%	92.44%	61.89%
White	93.86%	87.77%	94.79%
Black	4.19%	10.74%	3.19%
BMI	29.0 ± 5.64	27.4 ± 5.37	29.2 ± 5.64
Current smoking	7.27%	1.31%	8.18%
Pulse	74.3 ± 11.3	74.8 ± 10.8	74.2 ± 11.3
Systolic BP	133 ± 16.6	119 ± 11.6	135 ± 16.1
HTN Rx	53.33%	25.68%	57.53%
Aspirin use	43.54%	33.33%	45.09%
Any Statin use	44.79%	36.51%	46.05%
Ezetimibe	0.89%	0.56%	0.94%
Total cholesterol	190 ± 37.2	199 ± 37.7	188 ± 37.0
HDL-C	55.0 ± 15.6	60.5 ± 14.9	54.2 ± 15.5
LDL-C	108 ± 32.9	114 ± 33.8	107 ± 32.6
LDL≥190	1.77%	3.08%	1.58%
Diabetes	21.93%	0.37%	25.20%
Elixhauser index	2.09 ± 4.25	1.68 ± 3.87	2.15 ± 4.30
**Specialty seen**			
Cardiology	14.74%	9.99%	15.46%
PCP	85.26%	90.01%	85.54%

ASCVD indicates atherosclerotic cardiovascular disease; BMI, body mass index; BP, blood pressure; HDL-C, high-density lipoprotein cholesterol; HTN Rx, antihypertensive medication; Rx, medication prescription; LDL-C, low-density lipoprotein cholesterol; LDL, low-density lipoprotein; and PCP, primary care physician.

### 3.1 Outcomes

The high-risk group of older adults in this cohort who were on no statin had a significantly increased risk of MI [HR 1.51 (1.17–1.95); *p* < 0.01], stroke [HR 1.47 (1.14–1.90); *p* < 0.01] as well as all-cause mortality [HR 1.37 (1.19–1.58); *p* < 0.001] compared with those on a moderate or high intensity statin (Table 2).

**Table 2:** Hazard Ratios of Adverse Outcomes Stratified by ASCVD Risk and Statin Treatment in Older Adults ≥ 75 Years of Age.

Event	Intermediate risk (*n* = 1071)			High risk (*n* = 7043)		
	Moderate or High Intensity Statin	Low Intensity Statin	No Statin	Moderate or High Intensity Statin	Low Intensity Statin	No Statin
MI	*Ref*	0.67 (0.03–14.20)	1.09 (0.41–2.88)	*Ref*	0.94 (0.48–1.86)	1.51*(1.17–1.95)
Stroke	*Ref*	3.38 (0.95–12.00)	0.52 (0.20–1.32)	*Ref*	1.02 (0.54–1.95)	1.47*(1.14–1.90)
Mortality	*Ref*	1.62 (0.57–4.58)	1.35 (0.88–2.07)	*Ref*	1.04 (0.74–1.46)	**1.37**⁎⁎**(1.19–1.58)**

Data are presented as hazard ratios of MI (myocardial infarction), stroke (ischemic stroke or transient ischemic attack) and mortality across intermediate and high risk 10-year risk ASCVD categories. The 10-year ASCVD risk categories were defined as intermediate-risk (7.5%–19.9%), and high-risk (≥20%). Statins are defined as high intensity: Atorvastatin 40 and 80 mg, Rosuvastatin 20 and 40 mg; moderate intensity: Atorvastatin 10 and 20 mg, Rosuvastatin 5 and 10 mg, Simvastatin 20 and 40 mg, Pravastatin 40 and 80 mg, Lovastatin 40 and 80 mg; low intensity: Simvastatin 10 mg, Pravastatin 10 and 20 mg, Lovastatin 20 mg, Fluvastatin 20 and 40 mg. Data were fully adjusted for the Ellixhauser Comorbidity Index.

⁎ P value for trend <0.01.

⁎⁎ P value for trend <0.001. Abbreviations: ASCVD, atherosclerotic cardiovascular disease.

We did not find the same significance when comparing the high-risk cohort on a low intensity statin versus a moderate or high intensity statin (Table 2). When comparing the no statin group versus the moderate or high intensity statin group in the intermediate risk cohort, although a trend for increased risk was seen, it did not meet statistical significance thresholds for MI [HR 1.09 (0.41–2.88); *p* > 0.05), stroke [HR 0.52 (0.20–1.32); *p* > 0.05] or mortality [HR 1.35 (0.88–2.07); *p* > 0.05] (Table 2). HRs using no statin use as reference are shown in **Suppl. Table ii.**

In an explanatory analysis, we compared the risk of MI, stroke and mortality in the current cohort of older adults and those <75 years of age within the entire patient cohort described previously [12]. When compared to at least moderate statin use, no statin use was associated with a higher risk of MI [HR 1.48 (1.16–1.89)], stroke [HR 1.48 (1.15–1.89)] and all-cause mortality [HR 1.28 (1.12–1.47)]; all p values <0.01 (**Supplemental, Table iii a**). Data with no statin use as a reference are shown in **Supplemental, Table iii b**).

Further, we found that the probability of initiation of a statin therapy differed significantly between the age groups ≥75 years of age (in this study) versus those <75 years of age within our healthcare system at the time interval of two and five years (**Supplement Table I**). Amongst high-risk older adults, the probability of statin initiation of ‘moderate or high’ intensity was 20% at two years compared to ∼34% for those <75 years of age of equal risk (*p* < 0.001). At five years the probability of statin initiation of ‘moderate or high’ intensity was 36% for older adults compared to ∼57% for those <75 years of age of equal risk (*p* < 0.001).

Amongst intermediate risk older adults, the probability of statin initiation of ‘moderate or high’ intensity was ∼15% at two years compared to ∼24% for those <75 years of age of equal risk (*p* < 0.001). At five years the probability of statin initiation of ‘moderate or high’ intensity was ∼26% for older adults compared to ∼42% for those <75 years of age of equal risk (*p* < 0.001) (Supplement **Table i**).

## 4 Discussion

In this study of statin utilization and ASCVD outcomes in a primary prevention cohort of older adults between 75 and 79 years of age, stratified by 10-year PCE based risk, we present three key findings; 1) approximately 54% of high-risk and 64% of intermediate risk primary prevention older patients were not on any statin, 2) the probability that a statin would be initiated for these older patients remained low at 2 and 5 years (20% and 36% for the high-risk cohort and even lower for the intermediate risk cohort), and, 3) risk of ASCVD events including MI, ischemic stroke/TIA and mortality were significantly higher in the high-risk older individuals (≥75 years of age) who were on no statins compared to those on a moderate or high intensity statin (**Central Illustration**).

Limited evidence evaluating real-world data on statin use and ASCVD outcomes in a primary prevention cohort of older adults from large contemporary healthcare systems in the United States exists in literature.

Our study extends prior work by Sarraju et al., evaluated statin use for primary prevention in individuals 65–79 years of age and showed that older adults >75 years were less likely to receive a moderate or high intensity statin regardless of ASCVD risk compared to those who were <75 years [16]. Similar to our result these patients had a higher incidence of ASCVD events – followed up to only 1 year. In our current study we further explore statin use and outcomes including incident MI, ischemic stroke, and social security index confirmed all-cause mortality per the ACC/AHA 10-year risk in this cohort of older adults. Our results build on these prior data by showing a longer follow up of ∼ 7 years with a comprehensive capture of incident ASCVD events and mortality.

Previously a meta-analysis pooled data from several large cohort studies by Gencer et al., showed that lipid-lowering agents reduced cardiovascular events in older adults [17]. However, this and other studies have not included a breakdown of no statin use versus statin use of different intensities stratified by ASCVD risk in older adults (age ≥ 75 years) [12,[18], [19], [20], [21], [22]]. Our study differs in that we were able to use a real-world cohort with contemporary practice across several sites in an integrated healthcare system. Further, we assessed older adults intermediate and high-risk categories based on the pooled cohorts equation.

Statin use and associated hypercholesterolemia management for secondary prevention in older adults is significantly effective; however, despite these data, suboptimal utilization of statins in this vulnerable group of older adults is a well-known phenomenon [21,[23], [24], [25]]. Shown in our analyses, statin utilization is low among older adults for primary prevention. Medical provider inertia in using statins, due to myalgias and various factors in older adults is a well-documented phenomenon [26,27], however, based on our results, this intervention is likely needed given reduction in ASCVD events. While myalgias are a common reason for statin discontinuation or lack of initiation, it has been shown that many patients experience myalgias with similar frequencies whether on a statin or placebo indicating that this may be a psychological reaction [28]. Other barriers to prescribing statins in older adults are the risk of polypharmacy as well as present competing risks reducing life expectancy (such as cancer). A study focusing on the time to benefit of a statin used for primary prevention has shown that statins may help prevent ASCVD risk event if life expectancy is at least 2.5 years [29]. While there is a lack of clear data on the statin benefit in high-risk older adults >75 years, our work shows the statin utilization associated reduction in adverse events including MI, stroke and mortality is evident just the same as their younger counterparts. The benefit of statin use persisted despite competing comorbidities including cancer, dementia and chronic kidney diseases.

According to a recent survey, older patients have a higher risk-adjusted mortality with a great disability risk and have longer hospitalizations with incident cardiovascular disease risk [30]. Further, older adults receive less evidenced-based care, which may be one of the several underlying reasons for the increased hospitalizations and downstream healthcare burden [30,31]. The number of incident strokes is expected to more than double, with the majority of the increase amongst those >75 years of age [30]. With an aging population of the United States and longer life expectancy due to improvements in medicine it is crucial to ensure primary prevention interventions are offered where appropriate, to all patient populations. Statins are a cost-effective pharmacological option that is widely available and easily accessible. The lack of statin initiation in high risk older adults may be a lost opportunity for low-cost intervention to reduce ASCVD events and associated economic burdens.

Even though there is significantly increased events of MI, stroke and mortality in high-risk older adults on no statin, the probability that a statin will be initiated in 2 or 5 years in these individuals is very low (20% and 36% respectively) based on data from our study. This is further evidence that statin utilization in older adults is a missed opportunity that has potential for improvement. Currently there is limited comparative data on statin initiation in older adults however, trends of the limited data support that it is possible that the rates are low as we have shown given various practice model sites across the healthcare system [16].

The lack of statin use combined with low initiation rates may stem from unclear guidelines and unclear evidence on statin use in older adults (age > 75). Many major guidelines rely on having a discussion between the patient and provider about the initiation of a statin in individuals older than 75 years of age [1]. Lack of concrete guideline directions on statin use in older adults for primary prevention is likely due to the paucity of evidence and lack of recruitment of the aging adults in randomized controlled trials [27,31]. Our work can add to the literature to support clear guidelines for statin use among at least high-risk older adults.

It is likely that with on-the-horizon data from the PREVENTABLE (Pragmatic Evaluation of Events and Benefits of Lipid-Lowering in Older Adults) trial (Identifier: NCT04262206) and the STAREE (A Clinical Trial of Statin Therapy for Reducing Events in the Elderly) (Identifier: NCT02099123) will shed further insights on the risk-benefit of statin use in older populations.

### 4.1 Limitations

We recognize there are limitations present in this study. First, as an EHR was used for data collection this allows for inclusion of accidental inaccurate data or misclassified information to be included in the data collection [32]. Coronary artery calcium scores, which may reclassify ASCVD risk, and could have a potential use in initiation of statins were not included in this study due to limited data availability [11,33]. In addition, the study had a limited number of individuals in the older adult intermediate risk group as well as the Black race and those aged>79 years were excluded from the study due to inapplicability of the pooled cohort equations. Social determinants of health including healthcare insurance and patient annual income were not assessed. Furthermore, although there is potential selection bias involving a single healthcare network, our network comprises >35 hospitals encompassing approximately >200 outpatient clinics which account for variable practise types including academic as well as private and variable socioeconomic status. UPMC retains approximately 85% of its patient population for return care leaving a small percentage of patients seeking care from non-UPMC institutes where their ASCVD event rates could not be included. Statin dose changes and discontinuation could not be reliably assessed due to the EHR sampling.

## 5 Conclusion

In a large healthcare network, a majority of high-risk primary prevention older adults between 75 and 79 years of age were not on any statin and initiation remained low at 2 and 5 years. Lack of statin use was uniformly associated with increased MI, stroke and mortality in high-risk older adults over a period of seven year follow ups. Given the benefits appreciated with statin use in this older population, statin use may need to be more strongly considered for primary ASCVD prevention among high-risk older adults. Further studies will clarify the exact risk/benefit ratio of statin therapy use in older adults for primary prevention of ASCVD.

## Twitter summary

This study investigates statin utilization and cardiovascular outcomes in a real-world primary prevention cohort of older adults > 75 years of age.

## Research ethics statement

This study was approved by a Quality Improvement and Institutional Review Board committees. The informed consent requirement was waived for this study since it was restricted to secondary data analysis.

## Funding

No external funding for the current study.

## Disclosures

None.

## CRediT authorship contribution statement

**Aaron J. Walker:** Writing – review & editing, Writing – original draft. **Jianhui Zhu:** Writing – review & editing, Data curation. **Floyd Thoma:** Writing – review & editing. **Oscar Marroquin:** Writing – review & editing. **Amber Makani:** Writing – review & editing. **Martha Gulati:** Writing – review & editing. **Eugenia Gianos:** Writing – review & editing. **Salim S. Virani:** Writing – review & editing. **Fatima Rodriguez:** Writing – review & editing. **Steven E. Reis:** Writing – review & editing. **Christie Ballantyne:** Writing – review & editing. **Suresh Mulukutla:** Writing – review & editing, Writing – original draft, Conceptualization. **Anum Saeed:** Writing – review & editing, Writing – original draft, Conceptualization.

## Declaration of competing interest

We wish to confirm that there are no known conflicts of interest associated with this publication and there has been no significant financial support for this work that could have influenced its outcome.

## Appendix Supplementary materials

Image, application 1
