# Supplementary material for: Statin utilization and cardiovascular outcomes in a real-world primary prevention cohort of older adults
Source: Am J Prev Cardiol. 2024 Apr 5;18:100664. doi: 10.1016/j.ajpc.2024.100664 (PMC11043821; doi:10.1016/j.ajpc.2024.100664)
Supplement: Supplementary file 1 [file mmc1.docx]

**Supplemental Material**

Figure I: Primary Prevention Cohort Inclusion from the University of Pittsburgh Medical Center System

*Abbreviations: UPMC; University of Pittsburgh Medical Center, PCP; primary care physician (including outpatient internal medicine, general internal medicine and family medicine), CAD; coronary artery disease, CHF; congestive heart failure, ASCVD; atherosclerotic cardiovascular disease.*

**Supplemental Table i: KM Estimated Probability of Patients Starting GDSI (<75 Years of Age) Or At Least a Moderate Intensity Statin (≥ 75 Years of Age) After Enrolled**

| **ASCVD risk** | **Age** | **Probability at 2 years** | **Probability 5 years** |
| --- | --- | --- | --- |
| **Intermediate** | <75 | 23.6 (23.2-24.0) | 42.2 (41.7-42.7) |
|  | ≥75 | 14.7 (12.2-17.4) | 26.3 (22.9-30.0) |
| **High** | <75 | 33.7 (32.9-34.5) | 57.3 (56.3-58.2) |
|  | ≥75 | 20.0 (18.8-21.3) | 36.0 (34.4-37.7) |

The 10-year ASCVD risk categories were defined as intermediate-risk (7.5%–19.9%), and high-risk (≥20%). Probabilities are listed as percentages. GDSI for <75 years old is defined as moderate or high intensity for intermediate risk and high intensity for high calculated ASCVD risk by the pooled cohort equations.

*Abbreviations: ASCVD, atherosclerotic cardiovascular disease, GDSI; guideline directed statin intensity*

**Supplemental Table ii: Hazard ratios of outcomes stratified by ASCVD risk and statin treatment in older adults (*using no statin use as reference*)**

|  | **Intermediate risk** | | **High risk** | |
| --- | --- | --- | --- | --- |
| **Event** | Low Intensity Statin | Moderate or High Intensity Statin | Low Intensity Statin | Moderate or High Intensity Statin |
| **MI** | 0.61  (0.03 – 12.91) | 0.92  (0.35-2.42) | 0.66  (0.34-1.28) | 0.66  (0.51-0.86)** |
| **Stroke-TIA** | 6.55  (1.66-25.81)* | 1.94  (0.76-4.95) | 0.70  (0.37-1.33) | 0.68  (0.53-0.88) ** |
| **Mortality** | 1.20  (0.43-3.56) | 0.74  (0.48-1.14) | 0.76  (0.54-1.07) | 0.73  (0.64-0.84) ** |

***denotes statistically significant values with p<0.05**

****denotes statistically significant values with p<0.001**

**Supplemental Table iii a: Hazard Ratios of Adverse Outcomes Stratified by ASCVD Risk and Statin Treatment in Older Adults ≥ 75 Years of Age and Adults < 75 Years of Age**

| **Event** | **Intermediate risk (n=1,071)** | | | **High risk (n=7,043)** | | |
| --- | --- | --- | --- | --- | --- | --- |
|  | **Moderate/High Intensity Statin** | **Low Intensity Statin** | **No Statin** | **Moderate or High Intensity Statin** | **Low Intensity Statin** | **No Statin** |
| **MI** | ***Ref*** | 0.67  (0.04-11.64) | 1.15  (0.48-2.76) | *Ref* | 0.91  (0.46-1.79) | 1.48*  (1.16-1.89) |
| **Stroke** | ***Ref*** | 2.89  (0.83-10.07) | 0.52  (0.21-1.29) | *Ref* | 1.03  (0.54-1.95) | 1.48*  (1.15-1.89) |
| **Mortality** | ***Ref*** | 1.45  (0.52-4.06) | 1.48  (0.94-2.15) | *Ref* | 1.00  (0.72-1.40) | 1.28**  (1.12-1.47) |

*Data are presented as hazard ratios of MI (myocardial infarction), stroke (ischemic stroke or transient ischemic attack) and mortality across intermediate and high risk 10-year risk ASCVD categories. The 10-year ASCVD risk categories were defined as intermediate-risk (7.5%–19.9%), and high-risk (≥20%). Statins are defined as high intensity: Atorvastatin 40 and 80 mg, Rosuvastatin 20 and 40 mg; moderate intensity: Atorvastatin 10 and 20 mg, Rosuvastatin 5 and 10 mg, Simvastatin 20 and 40 mg, Pravastatin 40 and 80 mg, Lovastatin 40 and 80 mg; low intensity: Simvastatin 10 mg, Pravastatin 10 and 20 mg, Lovastatin 20 mg, Fluvastatin 20 and 40 mg. Data were fully adjusted for the Ellixhauser Comorbidity Index. * P value <0.01. ** P value <0.001.*

*Abbreviations: ASCVD, atherosclerotic cardiovascular disease.*

**Supplemental Table iii b. Hazard ratios of outcomes stratified by ASCVD risk and statin treatment in older adults (75 years and older) compared with <75 years (*using no statin use as reference*)**

| **ASCVD** | **Intermediate risk** | | **High risk** | |
| --- | --- | --- | --- | --- |
| **Event** | Low Intensity Statin | Moderate or High Intensity Statin | Low Intensity Statin | Moderate or High Intensity Statin |
| **MI** | 0.59  (0.03 – 10.08) | 0.87  (0.36 – 2.10) | 0.65  (0.33 – 1.26) | 0.68  (0.53 – 0.87) ** |
| **Stroke-TIA** | 5.54  (1.43-21.43)* | 1.92  (0.77-4.75) | 0.70  (0.36-1.32) | 0.68  (0.53-0.87) ** |
| **Mortality** | 1.02  (0.37-2.82) | 0.70  (0.47-1.06) | 0.78  (0.56-1.09) | 0.78  (0.68-0.89) ** |

*denotes statistically significant values with p<0.05

**denotes statistically significant values with p<0.001

References:

1. Saeed A, Zhu J, Thoma F, et al. Cardiovascular Disease Risk–Based Statin Utilization and Associated Outcomes in a Primary Prevention Cohort: Insights From a Large Health Care Network. *Circ Cardiovasc Qual Outcomes*. 2021;14.
